# Positioning the Value of Dietary Carbohydrate, Carbohydrate Quality, Glycemic Index, and GI Labelling to the Canadian Consumer for Improving Dietary Patterns

**DOI:** 10.3390/nu11020457

**Published:** 2019-02-22

**Authors:** Christopher P. F. Marinangeli, Joanna Castellano, Peg Torrance, Joanne Lewis, Carolyn Gall Casey, Jackie Tanuta, Julianne Curran, Scott V. Harding, David J. A. Jenkins, John L. Sievenpiper

**Affiliations:** 1 Nutrition Science and Regulatory Affairs, Pulse Canada. 920-220 Portage Avenue, Winnipeg, MB R3C 0A5, Canada; jtenuta@pulsecanada.com (J.T.); jcurran@pulsecanada.com (J.C.); 2 Q Quest. First Canadian Place, 100 King St W #5700, Toronto, ON M5X 1C7, Canada; jcastellano@qquestinc.com (J.C.); ptorrance@qquestinc.com (P.T.); 3 Diabetes Canada. 1400–522 University Ave, Toronto, ON M5G 2R5, Canada; Joanne.Lewis@diabetes.ca (J.L.); Carolyn.GallCasey@diabetes.ca (C.G.C.); 4 Department of Biochemistry, Memorial University, St. John’s, NL A1C 5S7, Canada; sharding@mun.ca; 5 Department of Nutritional Sciences, Faculty of Medicine, University of Toronto, Toronto, ON M5S 1A8, Canada; david.jenkins@utoronto.ca (D.J.A.J.); john.sievenpiper@utoronto.ca (J.L.S.); 6 Clinical Nutrition & Risk Factor Modification Centre, St. Michael’s Hospital, Toronto, ON M5B 1W8, Canada; 7 Division of Endocrinology & Metabolism, St. Michael’s Hospital, Toronto, ON M5B 1W8, Canada; 8 Li Ka Shing Knowledge Institute, St. Michael’s Hospital, Toronto, ON M5B 1T8, Canada

**Keywords:** glycemic index, carbohydrate quality, labelling, regulatory

## Abstract

The objectives of this qualitative study was to: (1) understand Canadian consumers’ knowledge and perception of dietary carbohydrates, carbohydrate quality, and the glycemic index (GI); and (2) determine Canadian’s receptiveness to GI labelling to assist with identifying and consuming foods of higher carbohydrate quality. Focus groups were recruited in Vancouver, Toronto, and Montreal and grouped according to body mass index (BMI) (NBW, normal body weight; PO, previously obese; and OW/OB, overweight/obese) and diagnosis with prediabetes and diabetes (PO (Vancouver) and OW/OB (Montreal and Toronto). Subjects in all groups linked excess consumption of carbohydrate with weight gain. PO and OW/OB groups were conflicted between perceived negative consequences and feelings of pleasure associated with carbohydrate consumption. Subjects were largely unfamiliar with the term ‘carbohydrate quality’, but were often associated with classifying carbohydrates as ‘good’ or ‘bad’. The concept of the GI resonated well across groups after exposure to corresponding educational materials. However, NBW groups largely felt that the GI was irrelevant to their dietary choices as they did not have a history of diabetes. PO and OW/OB groups associated the GI with diabetes management. The concept of a GI labelling program to help facilitate healthier carbohydrate choices was well received across all groups, especially when the low GI was interpreted as giving permission to consume foods they enjoyed eating. Results suggest that the GI could be used as a consumer-facing labelling program in Canada and assist with de-stigmatizing carbohydrate foods by helping to facilitate the consumption of carbohydrate foods that align with healthy dietary patterns.

## 1. Introduction

Nutrition science dictates that carbohydrates are elements of a healthy diet. However, over the last decade, carbohydrates have been scrutinized by media and consumers as having the potential to negatively affect human health. The negative perceptions around dietary carbohydrate stem from multiple factors, which include a lack of understanding of the role carbohydrates play in a healthy diet, and the transfer of misinformation from scientifically unsubstantiated sources. For example, consumers have increasingly antagonistic feelings toward dietary carbohydrate as a cause of weight gain [1]. Therefore, it is reasonable that the development of a credible and science-based communication tool could assist consumers to identify foods of higher carbohydrate quality and provide assurances around the role of carbohydrates as part of a healthy dietary pattern. The glycemic index (GI) is such a tool that could be positioned as a consumer-facing symbol of carbohydrate quality.

The GI of a food is generated in humans through a series of feeding interventions. It is calculated as the ratio of the incremental post-prandial blood glucose area under the curve of a test food to white bread or a liquid glucose bolus providing the same amount of available carbohydrate. Typically, these foods contain 50 g of available carbohydrates, but depending on the test food, 25 g is also sometimes appropriate [2]. The use of a common denominator (white bread or glucose) permits GI scores to be easily compared between foods. Generally, a food with a GI of ≤ 55 is considered to have a low GI [2]. Cut-offs of 56–69, and ≥ 70 are used to identify foods that have a medium and high GI, respectively [2,3]. These cut-offs are based on a glucose scale, either by direct measurement on a glucose scale, or conversion from a bread scale [2]. Given that, traditionally, post-prandial excursions in blood glucose levels are a target for the dietary management of diabetes, the GI has been positioned as a nutritional tool for people with prediabetes or diabetes to stabilize blood glucose levels after meals [4,5]. However, low GI diets have been associated with decreased risk of cardiometabolic risk factors for chronic disease [6]. This is of no surprise because many low GI foods are emphasized in dietary guidelines as healthy carbohydrates, including: whole grains, fruits and vegetables, legumes, and dairy. Thus, there is an opportunity for the GI to be used by consumers, with and without diabetes, to identify quality carbohydrate food that should be consumed more often by the general population.

The regulations and policies from the Canadian Food and Drug Regulations and Canadian Food Inspection Agency’s (CFIA) Guide for Food Labelling do not provide a framework or guidelines for the use of a GI symbol on food products. This is in contrast to Australia and New Zealand, where Schedule 4 of the Food Standards Code permits GI claims on foods so long as the Nutrient Profile Score is below a specific cut-off value [7,8]. The Glycemic Index Foundation in Australia provides a ‘low GI’ symbol program that can be used by food industry in Australia and New Zealand to assist consumers in identifying low GI foods. In fact, since its launch in Australia, The Glycemic Index Foundation’s GI Symbol Program has been successfully integrated into the Australian marketplace [9]. Although Canada’s regulatory framework for claims may not permit the use of the GI as a claim on food products at this time, third party identifiers that communicate the merits of food are permitted in Canada and could provide an avenue for pursuing GI labelling as a credible source of information to consumers.

Given their ongoing endorsement of the GI concept in therapeutic guidelines [5,10], in 2015, Diabetes Canada (formally the Canadian Diabetes Association) announced an initiative to develop a labelling framework that would allow industry to identify low GI foods in the labelling and advertising of their food to Canadian consumers. Thus, as a first step, an understanding of Canadian’s perceptions of carbohydrate and the GI concept is required. Using a qualitative research study design, the objective of this study was to understand Canadian consumers’ knowledge and perception of dietary carbohydrates, carbohydrate quality, and the GI. The second objective was to identify a strategy for positioning the GI as a consumer-facing labelling program that could assist with identifying carbohydrate foods of higher quality and their incorporation into healthy diets. 

## 2. Methods

### 2.1. Study Design

Focus groups were conducted in Vancouver, Toronto, and Montreal in July 2017. Phenotypic and health-based inclusion criteria for each consumer group is summarized in Table 1. Participants were recruited into three consumer segments: (1) normal body weight (NBW; BMI ≤ 24.9); (2) previously obese (PO; BMI ≤ 29.9); and (3) overweight/obese (OW/OB; BMI ≥ 25). While individuals in the PO group could have been classified as overweight (BMI >24.9 and ≤29.9), they represented a distinct group of individuals who had been able to achieve sustained weight loss for ≥2 consecutive years at the time that they were recruited for this study and placed them in a separate category from the OW/OB group.

In addition to Table 1, participants were 30 to 55 years of age, had a household income ≥$50,000 per year, were employed, and had completed at least a secondary level of education (e.g., high school diploma or equivalent). The educational cut-off aligns with the educational status of over 86% of the Canadian population between 25 and 64 years of age [11]. Subjects were excluded if they had participated in a focus group within the last six months, have participated in a focus group regarding weight gain or weight loss products, or identified themselves as “shy”, “cynical”, “hesitant”, “reserved”, or uncomfortable expressing themselves in a group setting. Participants were also excluded if they or a member of their household were employed in industries related to market research, public relations, media and/or advertising, fitness, or natural health products. To ensure similar familiarity with Canadian culture, which includes the food environment, subjects were also excluded if they had immigrated to Canada after eight years of age.

### 2.2. Focus Groups

**Figure 1:**
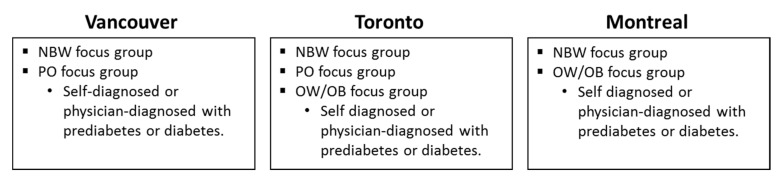
Summary of focus groups and consumer segments that took place in each city. Unless otherwise indicated, participants were not self- or physician-diagnosed with prediabetes or diabetes. NBW, normal body weight; PO, previous obese; OW/OB, overweight/ obese.

Two focus group sessions were conducted in each Vancouver and Montreal, while three sessions were conducted in Toronto (Figure 1). Focus groups in all three cities each included separate sessions with participants classified as NBW. Additional sessions in Vancouver and Montreal included individuals classified as PO and OW/OB, respectively. The two additional sessions in Toronto each recruited subjects characterized as PO and OW/OB. Subjects in the PO group in Vancouver and OW/OB groups Montreal and Toronto were physician-diagnosed or self-diagnosed with prediabetes or diabetes. Individuals in the PO group in Toronto were not diagnosed with pre-diabetes or diabetes. Focus group session ranged between 2.5–3 h in duration.

CRC Research Inc. (Vancouver, BC; Montreal, PQ, Canada) and Quality Response Inc. (Toronto, ON, Canada) recruited subjects for the focus groups in Vancouver and Montreal, and Toronto, respectively. In Vancouver and Toronto, J.C. (Joanna Castellano) served as the moderator. In Montreal, J.C. (Joanna Castellano) recruited, directed, and supervised a French speaking moderator. Within each city, the same moderator was used for each focus group. Furthermore, focus groups were conducted as semi-structured open-ended interviews in an isolated environment, and recorded for future analysis. Focus groups in Montreal were conducted in French and translated to English during the session.

A list of pre-defined objectives and questions were developed in advance and used by the moderator as a discussion guideline (Table 2, Parts 1–3). Focus groups were conducted in 3 segments. The first segment focused on the perception of carbohydrates and carbohydrate quality. The second segment explored participant’s familiarity with the GI and introduced educational materials from Diabetes Canada and the Glycemic Index Foundation (Figure S1). The third segment introduced participants to labelling concepts and promotional materials around the GI and was presented with the Glycemic Index Foundation’s GI Symbol Program used in Australia and New Zealand (Figure S2).

During each session, moderators probed individual experiences associated with carbohydrates, carbohydrate quality, and the GI. Where applicable, participants were asked to discuss the circumstances, feelings, and expectations within the context of the discussion. Following the completion of focus groups, transcripts were reviewed, coded, and interpreted by JC, PT, and CPFM to define perceptions and attitudes toward carbohydrates, carbohydrate quality, the GI, and GI labelling.

## 3. Ethics

This project was not reviewed by a research ethics board. CRC Research Inc. and Quality Response Inc. are consumer research firms and are Gold Seal members of the Market Research and Intelligence Association (MRIA), which conducts audits against defined standards for research methods, ethical practices, and personal privacy for agencies participating in consumer research [12]. As per Appendix E of the MIRA Code of Conduct for Market and Social Media Research, appropriate protections were in place to protect the privacy of subjects and attain informed consent. All subjects were screened by CRS Research Inc. and Quality Response Inc. and compensated $150–200 CAD for participating in the study.

## 4. Results

Subject demographics for all focus groups are summarized in Table 3. Forty-seven individuals across all focus groups participated in the study. More females (*n* = 40) were recruited compared to males (*n* = 7). Consequently, the majority of focus groups were comprised of solely female participants (NBW, Vancouver; NBW, Montreal; PO, Toronto; OW/OB, Toronto; OW/OB, Montreal). Also, the majority of participants (32%) were 30–34 years of age, followed by 50–55 years (21%), 35–39 years (19%), 40–44 years (17%), and 45–49 years (11%). Contrary to our inclusion criteria, one person from the Vancouver NBW group and two people from the Toronto PO group were > 25 and < 30 years of age; and four individuals in the Toronto OW/OB group were >55 and ≤ 60. Across the PO group in Vancouver and OW/OB groups in Toronto and Montreal, 65% of individuals had been diagnosed with prediabetes or diabetes by a physician, while the remaining 35% were self-diagnosed. Although the minimum income for participants was $50,000 CAD per year, one individual from the OW/OB group in Montreal had an income below this cut-off.

### 4.1. Defining and General Perceptions of Dietary Carbohydrate 

Across all groups, participants were able to identify dietary sources of carbohydrate. Examples ranged from sources of mono and disaccharides, such as “fruit”, “juice”, “sugar”, “candy”, and “corn syrup”, to sources of polysaccharide (starch and fibre) including, “breads”, “pasta”, “whole grains”, and “vegetables”. 

When the discussion focused on the contribution of carbohydrate to heathy diets and general health, opinions were similar within and among focus groups. Positive opinions about carbohydrates were often juxtaposed with the feeling that carbohydrates could have negative effects on health.

Participants within the NBW group demonstrated the most positive opinions toward dietary carbohydrate, where carbohydrates were not always linked to detriments in health, but were identified as healthy components of diets, and/or amongst their favourite foods to eat:

■ “I don’t worry about carbs” (NBW, Vancouver)
■ “I’ve never had a problem with carbs and weight gain. But as I get older I try to make my carbs count more.” (NBW, Vancouver)
■ “It (carbohydrate) does give you energy. If I don’t have carbs I do feel light-headed.” (NBW, Vancouver)
■ “Carbs are fundamental to the human diet. I don’t understand why, but they are required.” (NBW, Vancouver)
■ “Carbs are probably my favourite foods to eat. Probably the tastiest” (NBW, Vancouver)
■ “We need carbs. The good carbs those that are complex that are naturally occurring in the food. If I cut down too much it doesn’t work for me. But I want to avoid the white sugar, the refined sugar.” (NBW, Montreal)
■ “If you don’t have health problems you will have less of a tendency to watch this closely” (NBW, Montreal)

Within the NBW groups, weight gain was highlighted as the primary adverse effect that dietary carbohydrates can have on health and phenotype:

■ “Carbs and weight gain” (NBW, Vancouver)
■ “I’ve tried to cut out bread intake to lose weight” (NBW, Toronto)
■ “A lot of my friends are way healthier than I am. Gym buffs. That’s the first thing they tell me: Cut breads, pasta, and all that nonsense” (NBW, Toronto)

Despite perceived linkages between weight gain and carbohydrate, there was also a fondness for carbohydrate as foods that bring feelings of enjoyment and comfort:

■ “I’ve always associated it as an indulgence. Something that, if I eat too much, I will gain weight. It’s something that I feel like I’ve had to cut back on or not eat a lot of. Because I love it too much” (NBW, Vancouver)

For some, the conflict between enjoyment and possible ill effects on health elicited feelings of confusion, mistrust, and guilt when carbohydrates were consumed:

■ “Growing up, carbohydrates are the base of your diet... A lot of diet advice was low fat and fat is bad and use low fat diets to try to lose weight. Later on, you started to hear more information from research that having too much sugar in your diet and too many simple carbohydrates is a really bad thing. And you can have good fats and that is ok. It’s something where I originally had one idea about it (carbohydrate), but that has changed over the course of my life.” (NBW, Vancouver)
■ “There is guilt associated with carbs even though we haven’t been properly educated that carbs are needed as part of a proper diet.” (NBW, Vancouver)

Similar to the NBW groups, individuals in the PO groups expressed positive feeling toward dietary carbohydrate. However, fondness for carbohydrate was rooted in feelings of comfort, tradition, and family, rather than as components of healthy dietary patterns:

■ “I like the carbs.” (PO, Vancouver)
■ “Having bread and pasta with a group of friends is great.” (PO, Vancouver)
■ “Comforting–especially if you are eating food that you were brought up eating. Then you are told you can’t do that anymore. Psychologically it is very frustrating. Especially if you get together with family and there is that wonderful and comforting feast. It’s an emotional connection. You justify it. It is a part of the celebration connecting with friends and family.” (PO, Vancouver)

PO groups also tended to disclose that carbohydrates are metabolized to components that have negative health consequences and/or cause weight gain. Some participants also disclosed strategies for avoiding dietary carbohydrate:

■ “Perception of carbs being bad” (PO, Vancouver)
■ “I was told by doctor to stay away from all the ‘white’ stuff… those are carbs that convert into sugars.” (PO, Vancouver)
■ “I was always taught that carbs make you gain weight.” (PO, Toronto)
■ “I started eating healthier. Less fast food. I tried to cut down on breads and starches. For breakfast, I would just do an egg scramble and not eat the toast…” (PO, Toronto)
■ “I never eat bread either. That is a big thing. Cutting out bread and pasta makes a huge difference” (PO, Toronto)
■ “I went to Pinterest for a lot of recipes on how to lose weight, for meal plans that they suggest. They always said to cut out the carbs.” (PO, Toronto)

Given that participants in the PO group had both positive and negative opinions about dietary carbohydrates, similar to the NBW group, they demonstrated a sense of confusion around the role carbohydrates have in healthy dietary patterns. For some, it was acknowledged that the environment has largely influenced their opinion that carbohydrates are foods that should be limited in the diet. At the same time, participants in Vancouver acknowledged that carbohydrates, not only generate a sense of satisfaction and pleasure to meals, but also could have a functional role in athletic performance:

■ “It is engrained in us that carbs are not really good, but you really do need them. I said satisfying because I feel great after eating them.” (PO, Vancouver)
■ “Carbs are always looked upon as a bad thing. But then again, especially with athletes, you need the carbs. It’s not just athletes, everyone needs carbs. It’s that balance of good vs. bad.” (PO, Vancouver)

In the OW/OB groups (Toronto and Montreal), positive and negative opinions of carbohydrate were not as prolific as with other groups. Under the theme of health and wellbeing, sentiments around carbohydrates were negative, but were contrasted with the belief that carbohydrate foods are enjoyed during eating occasions:

■ “You can’t eat anything white. I love potatoes, I love rice, I love pasta. Potatoes and bread are the hardest things to get rid of.” (OW/OB, Toronto)
■ “I cut out a lot of my carbs.” (OW/OB, Toronto)
■ “We don’t have the choice” (OW/OB, Montreal)
■ “I have frustration for pasta. I love pasta. I am not happy about that.” (OW/OB, Montreal)

### 4.2. Perceptions of Dietary Carbohydrate: Word Association

When participants were asked to generate words to describe their opinions of dietary carbohydrate, results generally mirrored the verbal feedback reported previously (Table 4). Across groups, negative words or phrases used to describe carbohydrates focused on consumption of excess calories or weight gain. In the PO and OW/OB groups, there were some references to blood sugar levels, or being ‘compromised’, which was likely due to their exposure to education around the potential effects of carbohydrate on blood sugar management. Positive words and phrases used to describe carbohydrate generally focused on the hedonic experiences associated with carbohydrate foods. Words and phrases that describe feelings of comfort, family, and nostalgia were also prevalent across groups.

### 4.3. Familiarity with Carbohydrate Quality

Generally, all participants were unfamiliar with the term ‘carbohydrate quality’. For some individuals, the notion of ‘carbohydrate quality’ contradicted their fundamental beliefs that carbohydrates were not part of healthy dietary patterns. Individuals most resistive to the term ‘carbohydrate quality’ were from the OW/OB group:

■ “No idea” (OW/OB, Toronto)
■ “I don’t believe there is a quality of carbs.” (OW/OB, Toronto)
■ “Oxymoron” (OW/OB, Toronto)
■ “No such thing – sugar is sugar” (OW/OB, Toronto)
■ “A carb is a carb” (OW/OB Montreal)

While unfamiliar with the term, some individuals in all groups interpreted ‘carbohydrate quality’ as the segregation of carbohydrate foods that have positive or negative effects on health; based on the nutritional or health benefits of carbohydrate-containing foods. Moreover, some individuals simplified the concept of ‘carbohydrate quality’ by categorizing foods as ‘good’ and ‘bad’ carbohydrates. This ability to segregate demonstrated that, while positive attributes of carbohydrates were largely based on feelings of pleasure and comfort, there was some understanding that some carbohydrate-rich foods are healthy:

■ “High in carbs but also high in fibre” (NBW, Vancouver)
■ “I’ve never heard that term before. I assume it’s related to other nutritional value. I would be more interested in that. It feels more related to me.” (NBW, Vancouver)
■ “Donut vs. oatmeal” (NBW, Toronto)
■ “Fruits and vegetables are good quality” (NBW, Toronto)
■ “Whole grains” (NBW, Toronto)
■ “Difference between simple and complex carbs. The foods that contain the complex carbs will be better for your body.” (PO, Vancouver)
■ “Complex carb—brown rice vs. white rice.” (PO, Toronto)
■ “Sounds like good carb/bad carb.” (PO, Toronto)
■ “Good carb? Anything bleached is bad—white rice, white pasta.” (PO, Toronto)
■ “Whole wheat is better.” (PO, Toronto)
■ “We are taught that whole wheat is better, but it’s not.” (PO, Toronto)
■ “For me slow sugars would be good carbs, fast sugars would be bad carbs.” (OW/OB, Montreal)
■ “Orange the fruit is good, the juice is bad.” (OW/OB, Montreal)
■ “Everything that is slow is food that hasn’t been altered. Everything that is fast has been processed.” (OW/OB, Montreal)

### 4.4. Familiarity with the Glycemic Index

Generally, participants in the NBW group had a neutral perception of the glycemic index. While a formal definition was not provided, excerpts presented in Table 5 demonstrate that subjective definitions of the GI was related to the measurement of ‘blood sugar’ levels, levels of sugar in food, or a means for comparing carbohydrate-containing foods.

One person from the Toronto and Montreal NBW groups indicated that the GI was a “guide to healthy eating” and “choices for combining foods together”, respectively. For two individuals, the GI was related to illness and/or diabetes:

■ “Anyone who is sick needs to be closely monitored. Anyone with a health concern” (NBW, Vancouver)
■ “For diabetes” (NBW, Montreal)

Three individuals from the OW/OB group (Toronto) and 1 from PO group (Toronto) were unfamiliar with the GI. Some subjects in the PO and OW/OB groups also expressed negative feelings toward the GI because, to them, it was related to diabetes/disease, dieting, and sugar:

■ “Lists of different types of foods broken down into high sugars. Foods that have high sugar content vs. low. Gives me an indication as to which foods are better for me when it comes to low sugar and which are not as good for me because they have high sugar content.” (PO, Vancouver)
■ “Glycemic index is negative” (PO, Toronto)
■ “Associate with disease” (PO, Toronto)
■ “A way to catch yourself” (OW/OB, Toronto)
■ “You must follow this meal” (OW/OB, Toronto)
■ “Announces bad news” (OW/OB, Montreal)
■ “Sugar” (OW/OB Montreal)

Again, higher prevalence of negative feelings toward the GI in the PO and OW/OB groups could stem from previous diagnosis with prediabetes or diabetes, and/or exposure to educational materials on the glycemic index as a means to managing blood glucose levels.

### 4.5. Reaction to Educational Materials on the Glycemic Index

Educational materials from Diabetes Canada and the Glycemic Index Foundation (Figure S1) that explained the GI, and how it relates to carbohydrate foods and heath elicited positive responses across groups:

■ “I liked reading this. Information was easy to digest. I am still curious about who is funding this but I also trusted it was science to a degree.” (NBW, Vancouver)
■ “Made me understand everything else a little more.” (NBW, Vancouver)
■ “As I’m shopping I’m not looking through all the information.” (NBW, Vancouver)
■ “Easy to read” (NBW, Toronto)
■ “Easy to understand” (NBW, Toronto)
■ “Glycemic label would help me make better choices” (PO, Toronto)
■ “Would be helpful for people to know I think, especially if diabetes runs in the family.” (PO, Toronto)
■ “I really like this list. It tells you what’s good and what to not have as much of.” (OW/OB, Toronto)

For individuals in the NBW group, educational materials increased or confirmed participant’s understanding of the GI. Some individuals in the NBW groups indicated that they preferred the GI compared to terminology around ‘carbohydrate quality’ as a means of choosing carbohydrate foods:

■ “Confirms what I knew (about the glycemic index) and makes me want to pay more attention to grocery shopping and the foods that I consume.” (NBW, Vancouver)
■ “Talking about GI works. It’s easier to say ‘GI’ than carbohydrate quality.” (NBW, Vancouver)
■ “In our minds, carbohydrates was the same as sugar. But even with sugar, we know there are good sugars and bad sugars. These sugar rich foods in general have a low glycemic index. In my mind it is still the same thing so this is strange. You have to define for me what carbohydrate is vs. what the glycemic index is. We know what the glycemic index is, but we don’t know what carbohydrates are.” (NBW, Montreal)

Although, reactions to the materials were similar in the PO and OW/OB groups, these groups tended to discuss how the use of the GI could improve the quality of their diets and help them make better dietary choices to enhance health or manage diabetes. Furthermore, they indicated that the GI removed some stigma that is commonly associated with carbohydrate foods that they enjoy eating:

■ “This is really good. The information about GI. Under grains—pasta and noodles, I thought they would be medium. It surprised me to see that they were low.” (PO, Vancouver)
■ “I was surprised to see that the things we think are ‘healthy’ are ‘bad’.” (PO, Toronto)
■ “Pasta is good” (PO, Toronto)
■ “This is showing me I can have noodles and popcorn without feeling guilty - this whole thing is guilt ridden. I feel guilty for looking at a potato. Therefore, this is great and uplifting. It is a very dramatic thing to find out if you are diabetic.” (OW/OB, Toronto)
■ “Surprised that foods that are high carb, low glycemic foods.” (OW/OB, Montreal)

The use of tools that permit consumers to easily exchange low GI foods for high GI foods was particularly appealing across groups in Vancouver and Toronto:

■ “Replace chart was good too” (NBW, Vancouver)
■ “Don’t cut, just swap —something easier to do that can be maintained as a lifestyle.” (NBW, Toronto)
■ “Being diagnosed and then given a lot of information can be confusing and overwhelming. What I found helpful with this was the reassurance that you don’t have to cut out everything, and just swap. That was a positive message to reassure you that you don’t have to give up your diet. So that is a positive message.” (PO, Vancouver)
■ “Simple swaps” (OW/OB, Toronto)
■ “I’m excited about the swap thing. I know what I am going to swap with. I love it.” (OW/OB, Toronto)
■ “I don’t want to feel like I can’t have something. I like the idea of swaps.” (OW/OB, Toronto)

Some individuals in NBW group felt that they did not require the GI to make healthy carbohydrate choices, “I don’t need the glycemic index to tell me what is good or bad” (NBW, Toronto), or felt that the GI was a tool that would only be useful for people diagnosed with diabetes:

■ “If I saw this I wouldn’t pick up the pamphlet because I don’t have diabetes.” (NBW, Toronto)
■ “If it was Health Canada it would be more general than just diabetes.” (NBW, Montreal)
■ “It’s not that it doesn’t seem reliable, it doesn’t seem like its targeted to me.” (NBW, Montreal)
■ “The title is a medical term, the ‘glycemic index’, if it was in parentheses and you put a more general term, if I had seen ‘glycemic index’ I would think it is not for me.” (NBW, Montreal)

### 4.6. Reaction to Glycemic Index Labelling on Food Products

When presented with the documentation from the Glycemic Index Foundation and examples of the GI symbol being utilized on food products in the Australian and New Zealand marketplace (Figure S2), overall reactions across focus groups were positive and that the use of a GI symbol on foods could be useful for communicating the presence of healthy carbohydrate-containing foods to Canadians (Table 6). Negative reactions to a GI symbol on foods were disproportionally higher among NBW and PO groups from Vancouver. For the former, this was primarily due to the perception that a GI symbol would not present any new information that would facilitate a change in their food choices. In the PO group from Vancouver, participants were skeptical about the legitimacy of the GI symbol and the verification of foods as having a low GI.

Despite broad support and positivity toward the Glycemic Index Foundation’s GI Symbol, ownership, management, and scientific integrity of a GI symbol in the Canadian marketplace were questioned by participants. In addition, success of a similar program in Canada would be contingent on transparency, scientific rigor, and governed by a trusted organization:

■ “Low GI seal is on white rice, on white corn, and on honey. I would think of those as really processed white carbs. Honey is just sugar. That would make me doubt whether this seal is telling me something reputable or important. I would want to do more reading on what this seal meant.” (NBW, Vancouver)
■ “I’m always skeptical. I always look anyways, even if there is something on the front of the box. It doesn’t mean it is a guarantee.” (NBW, Montreal)
■ “If it’s a trusted foundation in Australia then that’s the most effective. I’m thinking if that was swapped for the CDA (Canadian Diabetes Association [Diabetes Canada]) then that would be effective for me because I trust CDA.” (PO, Vancouver)
■ “Certified by an organization” (OW/OB, Montreal)
■ “Connected to research that was done by researchers according to statistics” (OW/OB, Montreal)
■ “There are so many foods with so many logos. They use this as marketing. Sometimes it’s true, sometimes it’s not.” (OW/OB, Montreal)
■ “If there is an association certified by them, it would add credibility” (OW/OB, Montreal)
■ “Approved according to what criteria?” (OW/OB, Montreal)

## 5. Discussion

Results from this study demonstrate that Canadian’s perception of dietary carbohydrate can differ across demographic groups underpinned by body weight, history of obesity, and/or diagnosis of prediabetes or diabetes. Although, the notion ‘carbohydrate quality’ did not resonate as a marketing tool, focus groups did interpret ‘carbohydrate quality’ as the categorization of carbohydrate foods as either ‘good’ or ‘bad’. The concept of the GI as a facilitator of positive food choices resonated well across groups, especially when low GI foods would translate into permission to consume foods that they enjoyed eating. 

This study highlights the conflict between positive and negative attributes of carbohydrate. Positive reactions to carbohydrate rooted in comfort, tradition, and taste were especially prominent amongst the PO and OW/OB groups whereas some individuals from the NBW groups identified carbohydrates as components of a healthy diet. During discussions around ‘carbohydrate quality’, the ability for respondents to identify healthy carbohydrate foods, such as those with whole grains, fibre, and whole fruits and vegetables, contradicted their underlying beliefs that consumption of carbohydrate and/or their removal from the diet caused weight gain or weight loss, respectively. This belief persisted despite existing science demonstrating weak or inconclusive associations between carbohydrate intake and weight loss or weight gain [13,14,15]. This apparent contradiction was largely due to information on nutrition and dieting from peers and other influencers that has been scientifically unsubstantiated. These sources of information elicited strong negative feelings toward carbohydrate that overshadow dietary guidelines, which globally, promote their consumption.

In the present study, although participants subjectively distilled ‘carbohydrate quality’ to ‘good’ or ‘bad’ carbohydrate foods, there was a general lack of understanding as to what ‘carbohydrate quality’ meant for their personal health and wellbeing. For some, visceral reactions to carbohydrate demonstrated confusion and disbelief that the term had face validity. This was not surprising given that the concept of carbohydrate quality is relatively new within the scientific community and encompasses a variety of measures including dietary fibre, whole grains, and glycemic response and/or GI [16]. For example, studies from the SUN cohort have used a carbohydrate quality index (CQI) that included fibre intake, the GI, the whole grain-to-total carbohydrate ratio, and the solid carbohydrate-to-liquid carbohydrate ratio [17,18,19]. At a recent workshop at the International Life Sciences Institute North America in 2017, carbohydrate quality was identified as whole and intact foods (i.e., grains, pulses, fruit, vegetables, and diary), foods with a low GI or glycemic response, and/or carbohydrate foods that are a source of fibre [20]. However, it remains undetermined how these attributes of carbohydrate quality criteria would be applied and communicated to consumers to affect food choices.

Results from this study showed that initial reactions to carbohydrate quality terminology were negative or neutral. However, a program anchored in a single concept of carbohydrate quality is a strategy that could prevent confusion and facilitate widespread adoption. Nutrient content claims for dietary fibre is a regulatory tool and the most straightforward example for indicating that a food is a source of healthy carbohydrate. Similarly, industry tools, such as the Whole Grain Stamp from the Oldway’s Whole Grain Council is a front-of-pack labelling program to demonstrate that a food contains at least 8 g whole grains per serving and has been adopted by industry stakeholders [21]. In Australia, The Glycemic Index Foundation has been successful with initiating and managing the GI Symbol Program that permits food industry to easily identify foods with a low GI [9].

Participants in the present study demonstrated some familiarity with the GI, especially those in the PO and OB/OW groups. This could have been, in part, due to their diagnosis with prediabetes and diabetes and exposure to educational programs that emphasize low GI and low glycemic load foods [4,5,22]. Given its link to the management of diabetes across all groups, feelings toward the GI were, at times, negative because it was associated with restrictive eating practices required to adequately manage blood sugar levels. Many participants held the belief that healthy carbohydrates are not hedonically pleasing and perceived to exclude foods that were associated with feelings of comfort and enjoyment, such as pasta. When exposed to educational materials from Diabetes Canada and the Glycemic Index Foundation (Figure S1) that characterized foods as low GI and those foods that can be exchanged for low GI foods, participants in the NBW, PO, and OW/OB groups exhibited positive feelings toward the GI as a tool that gave permission to consume foods they enjoyed and decreased stress when choosing quality carbohydrate foods. 

As a recognized component of carbohydrate quality, a GI symbol program may also be a useful tool and guide for Canadian consumers. Many low GI carbohydrate foods are inherently higher in fibre, which can reduce levels of available carbohydrate. Given that some whole grain foods can facilitate high glycemic and insulinemic responses to foods [23], the GI could also be a useful metric for deciphering the carbohydrate quality of these foods [24]. Similar to dietary fibre and whole grains, low GI dietary patterns have been associated to reduced risk for diabetes (and management of diabetes), cardiometabolic diseases, and obesity [3,6,25,26,27]. Use of the GI as a guide for consuming quality carbohydrate could broaden the scope of healthy foods within a dietary pattern by also emphasizing legumes, vegetables, and fruits, which on their own can be significant sources of dietary fibre and are also linked to a decrease in cardiometabolic risk factors [28,29,30,31,32,33,34]. Furthermore, in the context of a low GI diet, pasta was also recently shown to not be associated with weight gain or adiposity [35]. 

Given that both fresh and processed foods can be sources of quality carbohydrate that have a low GI, similar to Australia and New Zealand, a GI symbol program in Canada could be used across a variety of food categories. The successful implementation of a GI labelling program would be contingent on transparency and trust, as well as consumer education. In the present study, despite positive reactions to the GI Symbol used in Australia and New Zealand, subjects in all focus groups were unfamiliar with the Glycemic Index Foundation, which raised concerns regarding the legitimacy of the program. Diabetes Canada was identified by some participants as a trusted organization that could manage such a program. However, this may have been related to exposure during the focus groups to educational materials on the GI that were developed by Diabetes Canada. Some individuals from the NBW groups indicated that, for them, the GI was irrelevant. Canadians with diabetes may serve as early adopters of a GI labelling program in Canada and further education and resources would be required to re-calibrate beliefs around GI so that it appeals to a broader consumer base. Augustin et al. [3] suggested that the association between low GI diets and heightened feelings of satiety, and possibly weight management, could be a relevant attribute of low GI diets. Furthermore, it is important to emphasize that a GI symbol program would give the consumer information that easily identifies low GI foods in the marketplace, and is not linked to a punitive requirement given ones health status.

While the development and implementation of GI labelling in Canada is feasible, it has been the subject of debate. Although Australia and New Zealand, and South Africa permit low GI and glycemic claims on foods, the European Food Safety Authority has rejected GI claims [36]. In the past, Health Canada’s reservations about GI labelling has stemmed from concerns that the measurement of the GI lacks the accuracy and precision required for food labelling, the GI does not consider the amount of a food consumed, and some foods with low GI values may not align with Canadian dietary guidelines [36]. Consequently, Health Canada has gravitated toward glycemic response claims, where, rather than a standard reference food of a glucose bolus or white bread, test foods are compared to a serving of similar foods [37]. However, concerns raised by Health Canada have been largely addressed by the International Carbohydrate Quality Consortium [38] and Wolever [24,39]. Implementation of a nutritional profiling system for eligible foods would prevent unhealthy foods from making a low GI claim. To make a low GI claim in Australia and New Zealand for example, the food must have a Nutrient Profiling Score below a specific threshold [8]. The Nutrient Profiling Scoring method is integrated into Schedule 4 of the Australia New Zealand Food Standards Code [8]. The Glycemic Index Foundation’s GI Symbol Program also outlines that the method for testing the GI of a food must accord with the International Organization for Standardization (ISO) for GI testing [40,41], meet nutritional criteria for fat, sodium, and fibre across food categories, and contain at least 7.5 g carbohydrate or be ≥ 80% carbohydrate per serving [41]. Although nutrient profiling has not been integrated into Canadian regulations, profiling criteria already limit levels of saturated fat, sugar, cholesterol for foods that make disease reduction or therapeutic health claims [42]. Thus, it is reasonable that an organization that implements and manages a GI symbol program in Canada could adopt similar principles. Analysis of front-of-pack labelling programs in Canada demonstrated that foods bearing nutrition-based symbols were no more nutritious than foods without symbols [43]. The authors suggested that profiling could help consumers more effectively use font-of-pack symbols to improve the quality of diets [43].

## 6. Conclusions

The present study demonstrated that, rather than ‘carbohydrate quality’, Canadians, independent of their health history, were receptive to a labelling program that identifies carbohydrate foods as having a low GI. However, given that it was perceived that low GI foods were for the management of diabetes, successful implementation of low GI labelling will require significant consumer education and adoption by industry. Given that the low GI is a component of carbohydrate quality, the GI could be used in conjunction with other indices of quality carbohydrate such as ‘source of fibre’ and the promotion of whole foods. Results from this study suggest that the GI could be used as a consumer-facing labelling program in Canada and assist with de-stigmatizing carbohydrate foods by helping to facilitate the consumption of carbohydrate foods that align with healthy dietary patterns.

## Figures and Tables

**Table 1:** Inclusion criteria for BMI and history of diabetes for each group

NBW Group (Vancouver, Toronto, Montreal)	PO Group (Vancouver, Toronto)	OW/OB Group (Montreal, Toronto)
Have always maintained a healthy body weight (BMI ≤ 24.9), Subjects not diagnosed with diabetes.	History of obesity (BMI ≥30), but have maintained a BMI ≤ 29.9 for at least two consecutive years at the time they were recruited for the study. Subjects with and without prediabetes or diabetes were recruited. Prediabetes or diabetes was self-diagnosed or diagnosed by a physician.	BMI ≥ 25.Subjects with prediabetes or diabetes were recruited. Prediabetes or diabetes was self-diagnosed or diagnosed by a physician.

NBW, normal body weight; PO, previously obese; OW/OB, overweight/obese.

**Table 2:** Discussion guideline used by moderators during focus groups.

**Part 1: Exploration of Consumer Perspectives and Experiences Around Carbohydrate and Carbohydrate Quality.**
What is a carbohydrate?
What does quality of carbohydrate mean? Are their criteria for carbohydrate quality?
Unprompted: explore ‘good’ versus ‘bad’ carbohydrates.
What makes a carbohydrate ‘good’ or ‘bad’?
How do subjects know if a carbohydrate is ‘good’ or ‘bad’? Where did they hear about the concepts of ‘good’ and ‘bad’ carbohydrates? [Probe for: whole food ingredients, ‘source of fibre’ and GI or glycemic response]
Activity: Word Association
Have subjects make a list of ‘good’ vs. ‘bad’ carbohydrate foods.
Discuss the functional health benefits of carbohydrate; beyond the notion of ‘good’ and ‘bad’. (Unprompted examples include energy, weight loss, less lethargic, and no crashing).
**Part 2: Exploration of Consumer Perspectives and Experiences with the Glycemic Index**
What is GI?
How did you learn about the GI?
What does low? Medium? High GI mean?
Are there any references to carbohydrate and the quality of carbohydrates GI?
What foods are associated with having a low, medium, or high GI?
What are subject’s current behaviors in seeking out low GI foods?
When are subject’s apt to seek out low GI Foods? Which eating occasions?
What are the trade-offs in making low GI food choices?
What benefits are subject’s seeking in their decision-making around GI?
Does low GI index relate to high carbohydrate quality?
Why is GI important to you?
What is ‘good’ or ‘bad’ about GI
What are subject’s current behaviors in seeking out low GI foods?
When are subject’s apt to seek out low GI Foods? Which eating occasions?
What are the trade-offs in making low GI food choices?
What benefits are subject’s seeking in their decision-making around GI?
Does low GI index relate to high carbohydrate quality?
Why is GI important to you?
What is ‘good’ or ‘bad’ about GI
What are the benefits associated with knowing the GI of a food over short term? And over the long term?
Explore expectations of the sensory experience around the different GI foods and low/high quality carbohydrates.
Does the sensory experience vary with the time of day and moments of consumption?
How does sensory and moments of consumption affect beliefs and behaviours around choosing low or high GI foods?
Activity: Introduce materials that discuss benefits of low GI foods (Figure S1).
How can GI be positioned as something that is ‘good for everyone’?
What attributes are the positive attributes of low GI foods?
Is there other language that consumers prefer to use when discussing GI or carbohydrate quality?
How are the concepts of GI and carbohydrate quality the same or different?
What will resonate most (GI or carbohydrate quality) with consumers? (Examples include blood sugar control, healthy carbohydrate, high fibre, low sugar).
**Part 3: Discussion Pertaining to Low GI Labelling Concepts**
Can a GI label or quality of carbohydrate label help shift beliefs and behaviours?
Specifically, does GI/GR or carbohydrate quality change opinions on food?
Activity: You will be assessing various concepts, ideas and labels that may appear online, on products and elsewhere. Evaluate and provide your own individual reactions to each concept, idea, and label ( Figure S2).
What do you personally resonate with most in this idea and/or logo and why? What is this concept communicating to you?
What do you find most compelling and why? What do you find least compelling and why?
On a personal level, how convincing is the concept/logo about using these products from a scale of 1 to 10, where 1 is not all and 10 is extremely convincing?
How important is having this logo on food products? Is it trustworthy and why? Credible? How is it persuasive to you?

**Table 3:** Summary of demographic data for the subjects that participated in the focus groups.

Criteria	NBW Group			PO Group		OW/OB Group		Total
	Vancouver	Toronto	Montreal	Vancouver	Toronto	Toronto	Montreal	
*n*	7	7	6	7	7	7	6	47
Gender (n)								
Male	0	3	0	4	0	0	0	7
Female	7	4	6	3	7	7	6	40
Age (*n*)								
30–34 years	4 *	5	0	0	6 *	0	0	15
35–40 years	3	1	2	0	1	0	2	9
40–44 years	0	1	3	1	0	1	2	8
45–49 years	0	0	0	2	0	1	2	5
50–55 years	0	0	1	4	0	5 *	0	10
Mean (SD)	34.7 ± 3.9	33.4 ± 34.8	42 ± 7.0	49.3 ± 3.8	31.6 ± 4.2	53.9 ± 7.1	42.2 ± 5.0	41.0 ± 9.4
Body weight (kg) (Mean ± SD)	57.9 ± 10.4	64.2 ± 9.7	57.2 ± 6.5	72.9 ± 17.3	67.3 ± 6.5	86.3 ± 16.6	83.6 ± 20.5	69.9 ± 16.5
BMI (Mean ± SD)	21.3 ± 2.2	22.2 ± 1.9	21.9 ± 2.3	25.6 ± 3.2	23.3 ± 2.2	32.9 ± 5.2	31.0 ± 4.8	25.5 ± 5.3
Waist circumference (inch) (Mean ± SD)	27 ± 1.8	28.9 ± 3.7	29.2 ± 2.3	33.3 ± 3.9	29.9 ± 2.2 ^†^	38.1 ± 2.5	38.2 ± 7.0	32.0 ± 5.4
Diabetes or prediabetes (Y/N)	N	N	N	Y	N	Y	Y	
Diagnosed	N/A	N/A	N/A	4	N/A	7	2	13
Non-diagnosed	N/A	N/A	N/A	3	N/A	0	4	7
Annual income (n)								
50–59K	0	1	1	2	3	2	3 *	12
60–69K	0	0	3	1	1	0	1	6
70–79K	2	1	0	0	1	0	0	4
80–89K	1	1	0	3	0	1	0	6
90–99K	2	1	0	0	1	2	0	6
> 100 K	2	3	2	1	1	2	2	13
Marital status								
Married/common law	2	5	3	5	3	4	5	27
Single/separated/divorced	5	2	3	2	4	3	1	20
Education								
Completed secondary	1	0	3	2	3	2	1	12
Completed post-secondary	6	7	3	5	4	5	5	35

BMI, body mass index; N, no; N/A, not applicable; Y, yes. * Some individuals recruited exhibited characteristics that were outside the parameters of the inclusion criteria: One person from the Vancouver NBW and two people from the Toronto PO group were > 25 and < 30 and years of age; four individuals in the Toronto OW/OB group were >55 and ≤ 60; and one subject from the OB/OW Montreal group had an annual salary of < $50,000. ^†^ One subject in the Toronto PO group did not provide their waist circumference. The data point was imputed as the average waist circumference of the other six individuals within the same focus group.

**Table 4:** Results of the word association exercise for ‘carbohydrate’.

Negative Word Association	Positive Word Association
*NBW Group*	
Vancouver: “bad”, “calories”, “overeating and feeling heavy”, “controversy”, “empty calories”, “weight gain” Toronto: “crashing”, “weight gain” Montreal: “calories”, “weight gain”	Vancouver: “craving and comfort food”, “gives you pleasure” Toronto: “tastes good, good feelings, cravings”, “bad carbs brings me back to happy memories”, “loving and warm”, “enjoyment now” Montreal: “energy”, “a boost that allows you to be awake”, “allows you to work”
*PO Group*	
Vancouver: “good and bad”, “weight gain”, “bad for you”, “confusion”, “negative because it raised my blood sugar”, “sluggish” Toronto: “fat”, “heavy”, “bad but good”, “guilty”	Vancouver: “thirst quenching”, “energy”, “athletes”, “bursts”, “sports” Toronto: “grandma”, “family”, “delicious”, “tradition”, “carbs are associated with our background and our culture”, “comfort food”, “filling”, “energy”
*OW/OB Group*	
Toronto: “wrong”, “compromised”, “hidden” Montreal: “diabetic coma”	Toronto: “a staple”, “comfort food”, “filling”, “tasty”, “soothing”, “socially acceptable”, “my mom’s house”, “grandma’s house”, “enjoyable”, “favourite food” Montreal: “gives you energy”, “something we find on labels”

NBW, normal body weight; PO, previously obese; OW/OB, overweight/obese.

**Table 5:** Summary of subjective definitions of the glycemic index.

NBW Group	PO Group	OW/OB Group
Vancouver: ■ “How much sugar is in the item.” ■ “I think it’s for diabetic – they have to monitor it…has something to do with insulin regulating and how much sugar is in different foods.” ■ “It has to do with complex carbohydrates and sugars … and not having major spikes in blood sugar.” ■ “A high glycemic index is more simple sugars or things that you breakdown quicker…it will spike your blood sugar faster.” ■ “A low glycemic index body will process carbohydrates more slowly.” ■ “Is a scale and the higher you go, the faster your body changes what you are eating into sugar, you might have a sugar spike. A lower glycemic my understanding is that it is more of a plateau.”	Vancouver: ■ “Lists of different types of foods broken down into high sugars. Foods that have high sugar content vs. low. Gives me an indication as to which foods are better for me when it comes to low sugar and which are not as good for me because they have high sugar content.” ■ “Not the amount of sugar, but how quickly it converts to sugar in your system.” ■ “A scale or a chart. When I got diabetes, I got sent to the diabetes centre. I think of the chart that they have with high, med and low and certain foods fall under that. Honestly, I did that in 2015. I remember a few things. I don’t remember what goes where.” ■ “At the top of my head no. Some things get into your bloodstream and create that ‘surge’. In the time I’ve had diabetes, I’ve never felt a real ‘surge’. I don’t know if people feel that or not.” ■ “Glycemic index is synonymous with sugar, with carbs its food in general.” ■ “Glycemic index is an umbrella for which carbs fall under.” ■ “Glycemic index is not food, whereas carbohydrate is food.”	Toronto: ■ “It’s how your body is burning that particular food and how that food has a sugar ratio in it.” ■ “People who are type 1 diabetics, it’s the way they describe it to me.” ■ “The way certain sugars are broken down in the body.”
Toronto ■ “Comparing simple carbohydrates to high carbohydrates.” ■ “Foods with low glycemic index – I can eat as much as I want.” ■ “I feel like this would help me for sure.” ■ “ I never knew what it was.” ■ “A guide to healthier eating.”	Toronto ■ “Measurement of glucose in your body—your blood sugar level.” ■ “Can’t remember what it is but associate it with something to do with sugar.” ■ “A scale of some sort.”	
Montreal ■ “Measures the level of sugar in your blood.” ■ “You can have the glycemic index of a food.”		Montreal ■ “Control-controlling your blood sugar.” ■ “Glycemic levels overall, not just sugar, the level of carbs.” ■ “Blood tests.”

NBW, normal body weight; PO, previously obese; OW/OB, overweight/obese.

**Table 6:** Summary of reactions to the Glycemic Index Foundation’s GI Symbol that is used on food products in Australia and New Zealand.

Group	Positive Reactions	Negative Reactions
**NBW Group**	“I would pay attention to a low GI number because what I learned about it a low GI number was better for me.” (NBW, Vancouver) “To me that (GI) trumps carbs because we need carbs and the GI index is a way of saying whether it is a complex or simple carb. It is more of an indicator. To me, carb doesn’t tell me as much information.”(NBW, Vancouver) “Packaging is colourful and bright. Even if I didn’t know about GI, I would associate it with positive things” (NBW, Vancouver) “Must mean that it is under a certain level, whatever GI is. Obviously the lower the GI the better for you. I still don’t know what it means, but the lower the better” (NBW, Vancouver) “I like the idea” (NBW, Toronto) “I love it. Shows me what is good about this (food)” (NBW, Toronto) “I don’t have to cut carbs out” (NBW, Toronto) “Guide when I’m grocery shopping to make things easier and quicker” (NBW, Toronto) “It’s not just about eating protein” (NBW, Toronto) “Means that its certified, that it is low” (NBW, Montreal) “At the grocery store, if I knew what this was and I was informed, and I saw one loaf of bread with a stamp and one without. I would take the one with the stamp because then I know that it is low glycemic. But we are not diabetes, we just want to avoid it. We have to be informed ahead of time. If you know it will inform your choice” (NBW, Montreal) “I think it would be a good thing. It would make things easier” (NBW, Montreal) “It allows to make better choices” (NBW, Montreal) “It has to come with information. If I hadn’t seen it on TV before or somewhere, it would say nothing to me. There has to be a campaign” (NBW, Montreal) “With a foundation, it would be better” (NBW, Montreal) “Certified is stronger in my mind” (NBW, Montreal)	“No because I don’t know enough about it” (NBW, Vancouver) “It’s not something to worry about. I’ve never noticed any bad health effects from eating one or the other” (NBW, Vancouver) “We generally know the difference between good and bad food, but we don’t categorize that as high GI or low GI.” (NBW, Vancouver) “If I had a weight problem, I think I would be researching it more” (NBW, Vancouver) “If I saw it I probably wouldn’t pay attention because, to me, it would still be a calorie and fat. The only way I would be if the store had a marketing campaign and tell me why I need to know this” (NBW, Vancouver) “Doesn’t do anything for me because I’m uneducated on what GI means” (NBW, Vancouver)
**PO Group**	“This is for overall health” (PO, Toronto) “Easy to explain” (PO, Toronto) “Grabs your attention” (PO, Toronto) “Stamp means it’s healthier for you” (PO, Toronto) “I would look at the logo and associate it with a “good thing” (PO, Toronto) “Certified makes it look like it’s a good thing” (PO, Toronto) “Use QR codes or website to provide information on what low GI is” (PO, Toronto)	“Gimmicky. Having that in front of me doesn’t change anything. Honey has a low GI thing and it’s sugar” (PO, Vancouver) “ “Certified and foundation” sound too much like a phrase or trying to hard” (PO, Vancouver) “I don’t like the wording, I don’t trust it. I don’t know what they are” (PO, Vancouver) “A glycemic index label means nothing at this point” (PO, Toronto)
**OW/OB Group**	“Would be good, would be easy, nobody has time to read all the labels” (OW/OB, Toronto) “Inform me so I can make the right decisions” (OW/OB, Toronto) “You can eat healthy, here are the options” (OW/OB, Toronto) “It’s fine. I would definitely pick one over the other if I see that” (OW/OB, Toronto) “Makes grocery shopping easier” (OW/OB, Toronto) “I like knowing what I’m supposed to buy” (OW/OB, Toronto) “Help you figure it out” (OW/OB, Toronto) “There is food that I can eat and don’t need to go all over the place to figure it out” (OW/OB, Toronto)	“I don’t trust it which foundation authorizes it—they lie all the time” (OW/OB, Toronto) “Don’t say it’s certified” (OW/OB, Toronto)

## Supplementary Materials

The following are available online at https://www.mdpi.com/2072-6643/11/2/457/s1, Figure S1: Supplementary Appendix 1 A–C; Figure S2: Supplementary Appendix 2A–C.
