# Supplementary material for: Positioning the Value of Dietary Carbohydrate, Carbohydrate Quality, Glycemic Index, and GI Labelling to the Canadian Consumer for Improving Dietary Patterns
Source: Nutrients. 2019 Feb 22;11(2):457. doi: 10.3390/nu11020457 (PMC6412396; doi:10.3390/nu11020457)
Supplement: Supplementary file 1 [file nutrients-11-00457-s001.pdf]

Supplementary Appendix 1 A-C

Positioning the Value of the Dietary Carbohydrate, Carbohydrate Quality, the Glycemic Index, and GI Labelling to the Canadian Consumer for Improving Dietary Patterns

Christopher P.F. Marinangeli PhD, RD[1], Joanna Castellano,[2] Peg Torrance,[2] Joanne Lewis RD,[3] Carolyn Gall Casey,[3] Jackie Tanuta,[1] Julianne Curran PhD,[1] Scott V. Harding PhD,[4] David JA Jenkins MD PhD,[5-8] John L. Sievenpiper MD PhD[5-8]

[1]Pulse Canada. 920-220 Portage Avenue, Winnipeg MB, R3C 0A5, Canada; [2]Q Quest. First Canadian Place, 100 King St W #5700, Toronto, ON, M5X 1C7, Canada; [3]Diabetes Canada. 1400 - 522 University Ave, Toronto, ON M5G 2R5, Canada; [4]Department of Biochemistry, Memorial University, St. John's NL A1C 5S7, Canada; [5] Department of Nutritional Sciences, Faculty of Medicine, University of Toronto, Toronto, Canada; [6] Clinical Nutrition & Risk Factor Modification Centre, St. Michael's Hospital Toronto, Canada; [7]Division of Endocrinology & Metabolism, St. Michael's Hospital, Toronto, Canada; [8]Li Ka Shing Knowledge Institute, St. Michael's Hospital, Toronto, Canada

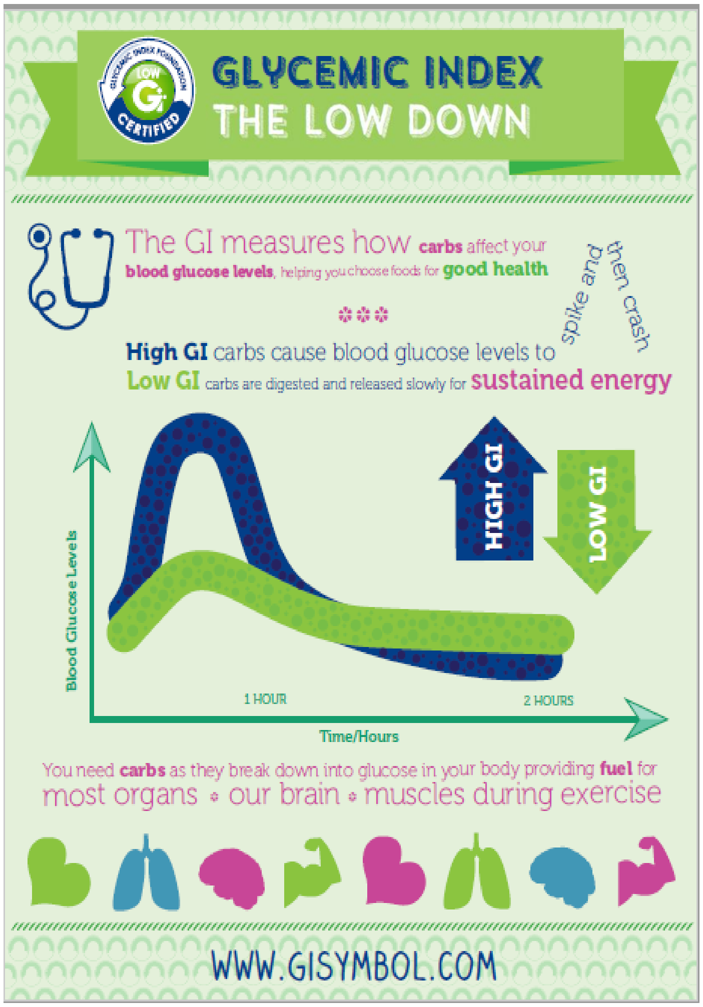

Supplementary Appendix 1A. Figure depicting the glycemic index (Adapted from the Glycemic Index Foundation[1]) .

The
Glycemic Index

Canadian Diabetes Association

What is the Glycemic Index of food?

The Glycemic Index (GI) is a scale that ranks carbohydrate-rich foods by how much they raise blood glucose levels compared to a standard food. The standard food is glucose or white bread.

Why should I eat foods with a low Glycemic Index?

Eating foods with a low Glycemic Index may help you to:

- Control your blood glucose level
- Control your cholesterol level
- Control your appetite
- Lower your risk of developing heart disease
- Lower your risk of developing type 2 diabetes

Use these meal planning ideas to include the Glycemic Index as part of healthy eating.

- Enjoy vegetables, most fruits and low-fat milk products with your meals. These are carbohydrate-rich foods that, in general, have low glycemic index.
- Plan your meals with foods in the low and medium Glycemic Index starch choices on the list that follows.
- Try foods such as barley, bulgar, or lentils, which have a low Glycemic Index.
- Consult a registered dietitian for help with choosing low GI foods, adapting recipes, and other ways to incorporate low GI foods in your meal plan.

If I eat foods with a low Glycemic Index can I eat as much as I want?

No. Using the Glycemic Index to choose foods is only one part of healthy eating.
Healthy eating also means:

- Eating at regular times
- Choosing a variety of foods from all food groups
- Limiting sugars and sweets
- Reducing the amount of fat you eat
- Including foods high in fibre
- Limiting salt

Remember that checking your blood glucose before and 2 hours after a meal is the best way to know how your body handles the meal.

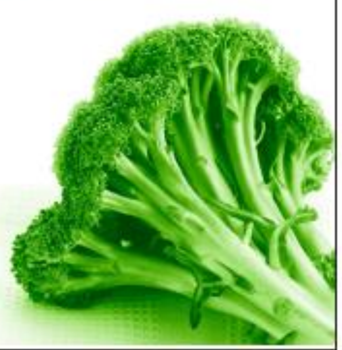

*This document reflects the 2013 Canadian Diabetes Association Clinical Practice Guidelines. ©2013 Copyright

A lot of starchy foods have a high Glycemic Index (GI). Choose medium and low GI foods more often.

LOW GI (55 or less) * † Choose most often	MEDIUM GI (56-69) * † Choose more often	HIGH GI (70 or more) * † Choose less often
**BREADS:**<br>100% stone ground whole wheat<br>Heavy mixed grain<br>Pumpernickel	**BREADS:**<br>Whole wheat<br>Rye<br>Pita	**BREADS:**<br>White bread<br>Kaiser roll<br>Bagel, white
**CEREAL:**<br>All Bran™<br>Bran Buds with Psyllium™<br>Oat Bran™	**CEREAL:**<br>Grapenuts™<br>Puffed wheat<br>Oatmeal<br>Quick oats	**CEREAL:**<br>Bran flakes<br>Corn flakes<br>Rice Krispies™
**GRAINS:**<br>Barley<br>Bulgar<br>Pasta/noodles<br>Parboiled or converted rice	**GRAINS:**<br>Basmati rice<br>Brown rice<br>Couscous	**GRAINS:**<br>Short-grain rice
**OTHER:**<br>Sweet potato<br>Yam<br>Legumes<br>Lentils<br>Chickpeas<br>Kidney beans<br>Split peas<br>Soy beans<br>Baked beans	**OTHER:**<br>Potato, new/white<br>Sweet corn<br>Popcorn<br>Stoned Wheat Thins™<br>Ryvita™ (rye crisps)<br>Black bean soup<br>Green pea soup	**OTHER:**<br>Potato, baking (Russet)<br>French fries<br>Pretzels<br>Rice cakes<br>Soda crackers

*expressed as a percentage of the value for glucose
† Canadian values where available

Adapted with permission from: Foster-Powell K, Holt SHA, Brand-Miller JC. International table of glycemic index and glycemic load values Am J Clin Nutr. 2002;76:5-56

Across the country, the Canadian Diabetes Association is leading the fight against diabetes by helping people with diabetes live healthy lives while we work to find a cure. Our community-based network of supporters help us provide education and services to people living with diabetes, advocate for our cause, break ground towards a cure and translate research into practical applications.

*This document reflects the 2013 Canadian Diabetes Association Clinical Practice Guidelines. ©2013 Copyright

Canadian Diabetes Association

***Related articles:** just the basics, fibre and diabetes, sugars and sweeteners*

diabetes.ca | 1-800 BANTING

[illegible]

Supplementary Appendix 1B. Infographic from the Diabetes Canada (formally the Canadian Diabetes Association) used to educate patients on the GI [2].

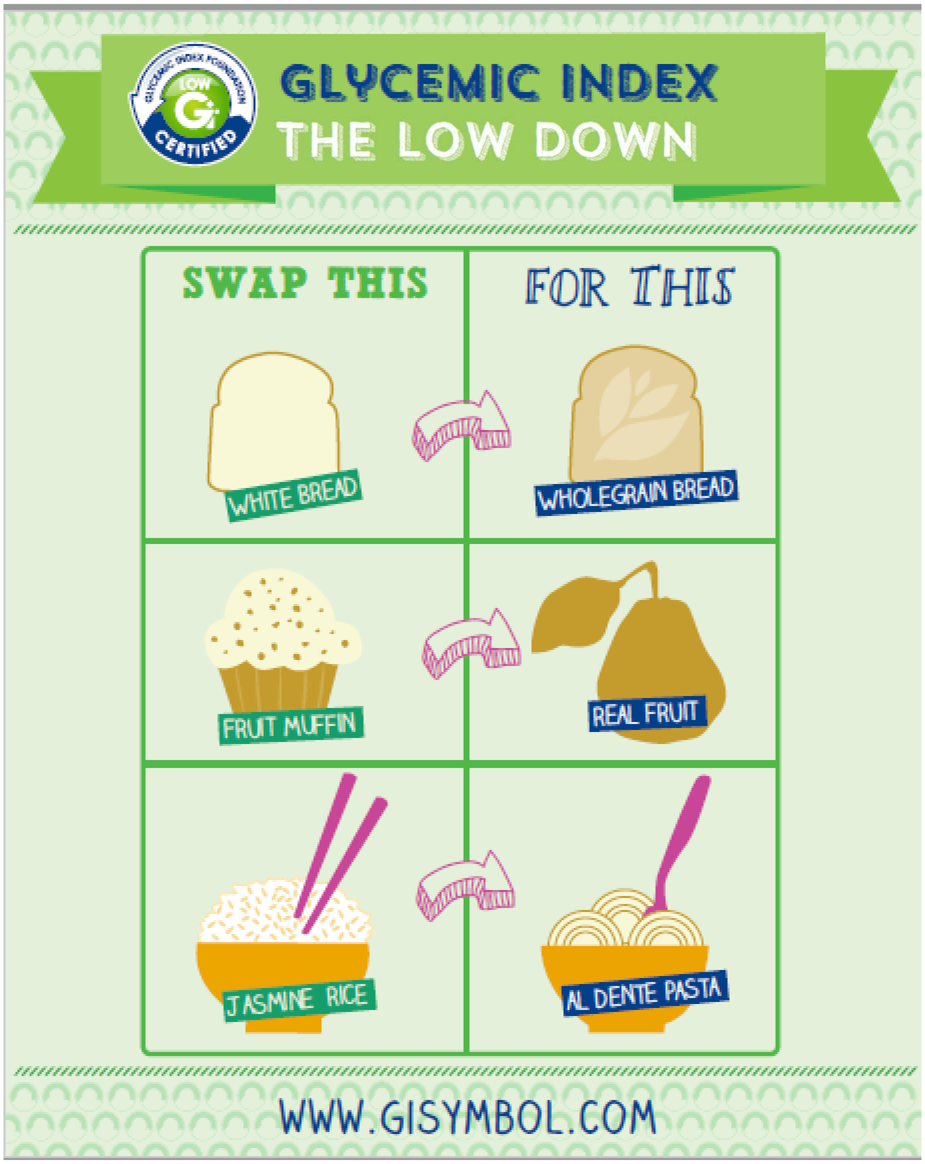

Supplementary Appendix 1C. Depiction of strategies for choosing low GI foods instead of high GI foods (Adapted from the Glycemic Index Foundation [3]).

References

1. The Glycemic Index Foundation. Glycemic Index: What is GI. The Glycemic Index Foundation. https://www.gisymbol.com/infographics/what-is-gi-infographic/ (October 10, 2018).

2. Diabetes Canada. The Glycemic Index. Diabetes Canada: Toronto, Ontario, 2013.

3. The Glycemic Index Foundation. Glycemic Index: Swap It. The Glycemic Index Foundation. https://www.gisymbol.com/infographics/swap-it-infographic/ (October 10, 2018).

Supplementary Appendix 2A-C

Positioning the Value of the Dietary Carbohydrate, Carbohydrate Quality, the Glycemic Index, and GI Labelling to the Canadian Consumer for Improving Dietary Patterns

Christopher P.F. Marinangeli PhD, RD[1], Joanna Castellano,[2] Peg Torrance,[2] Joanne Lewis RD,[3] Carolyn Gall Casey,[3] Jackie Tanuta,[1] Julianne Curran PhD,[1] Scott V. Harding PhD,[4] David JA Jenkins MD PhD,[5-8] John L. Sievenpiper MD PhD[5-8]

[1]Pulse Canada. 920-220 Portage Avenue, Winnipeg MB, R3C 0A5, Canada; [2]Q Quest. First Canadian Place, 100 King St W #5700, Toronto, ON, M5X 1C7, Canada; [3]Diabetes Canada. 1400 - 522 University Ave, Toronto, ON M5G 2R5, Canada; [4]Department of Biochemistry, Memorial University, St. John's NL A1C 5S7, Canada; [5] Department of Nutritional Sciences, Faculty of Medicine, University of Toronto, Toronto, Canada; [6] Clinical Nutrition & Risk Factor Modification Centre, St. Michael's Hospital Toronto, Canada; [7]Division of Endocrinology & Metabolism, St. Michael's Hospital, Toronto, Canada; [8]Li Ka Shing Knowledge Institute, St. Michael's Hospital, Toronto, Canada

Supplementary Appendix 2A. The Glycemic Index Foundation's Low GI Symbol [1].

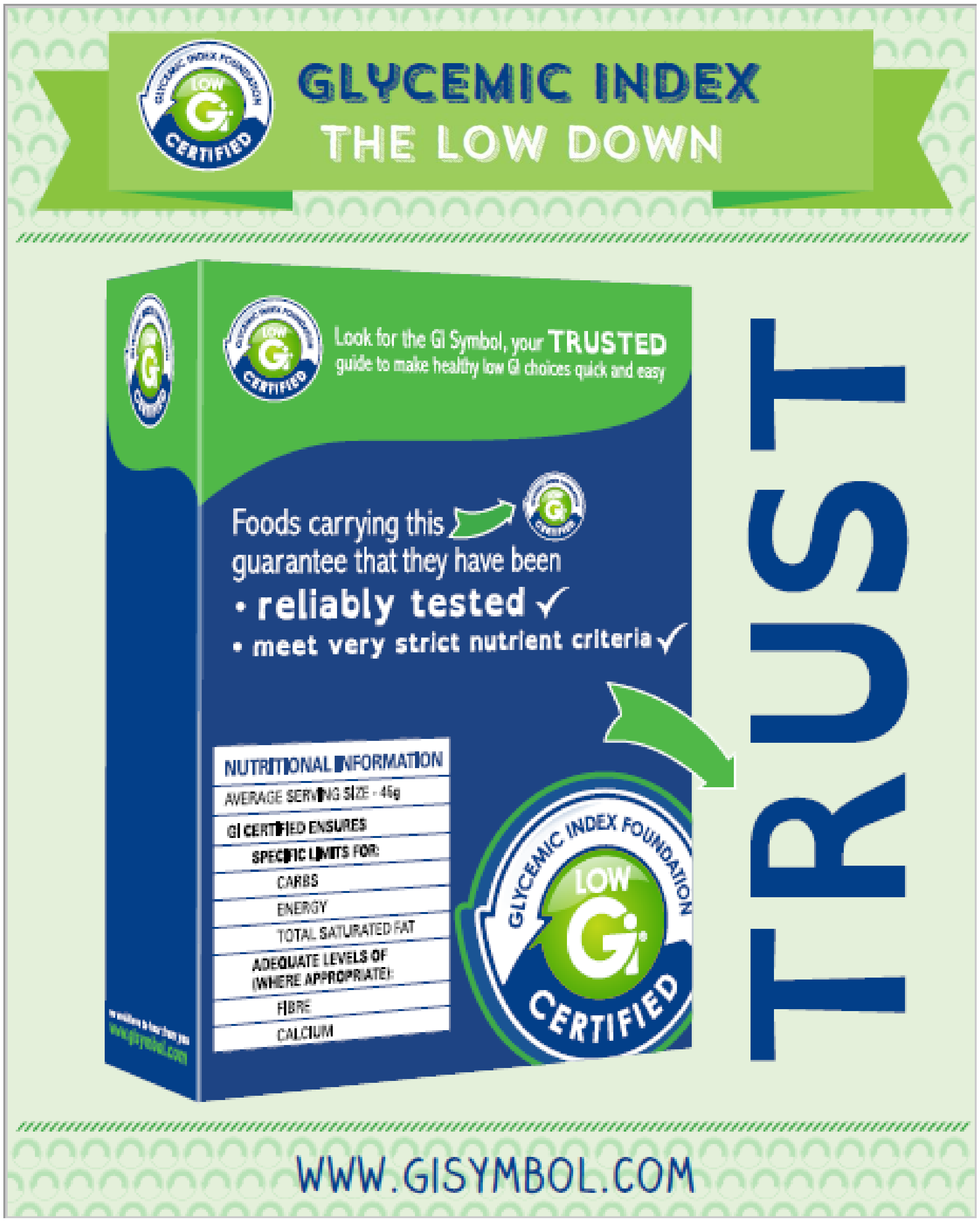

Supplementary Appendix 2B. Figure depicting the use of the Glycemic Index Foundations GI Symbol on a hypothetical food product (Adapted from The Glycemic Index Foundation [2]).

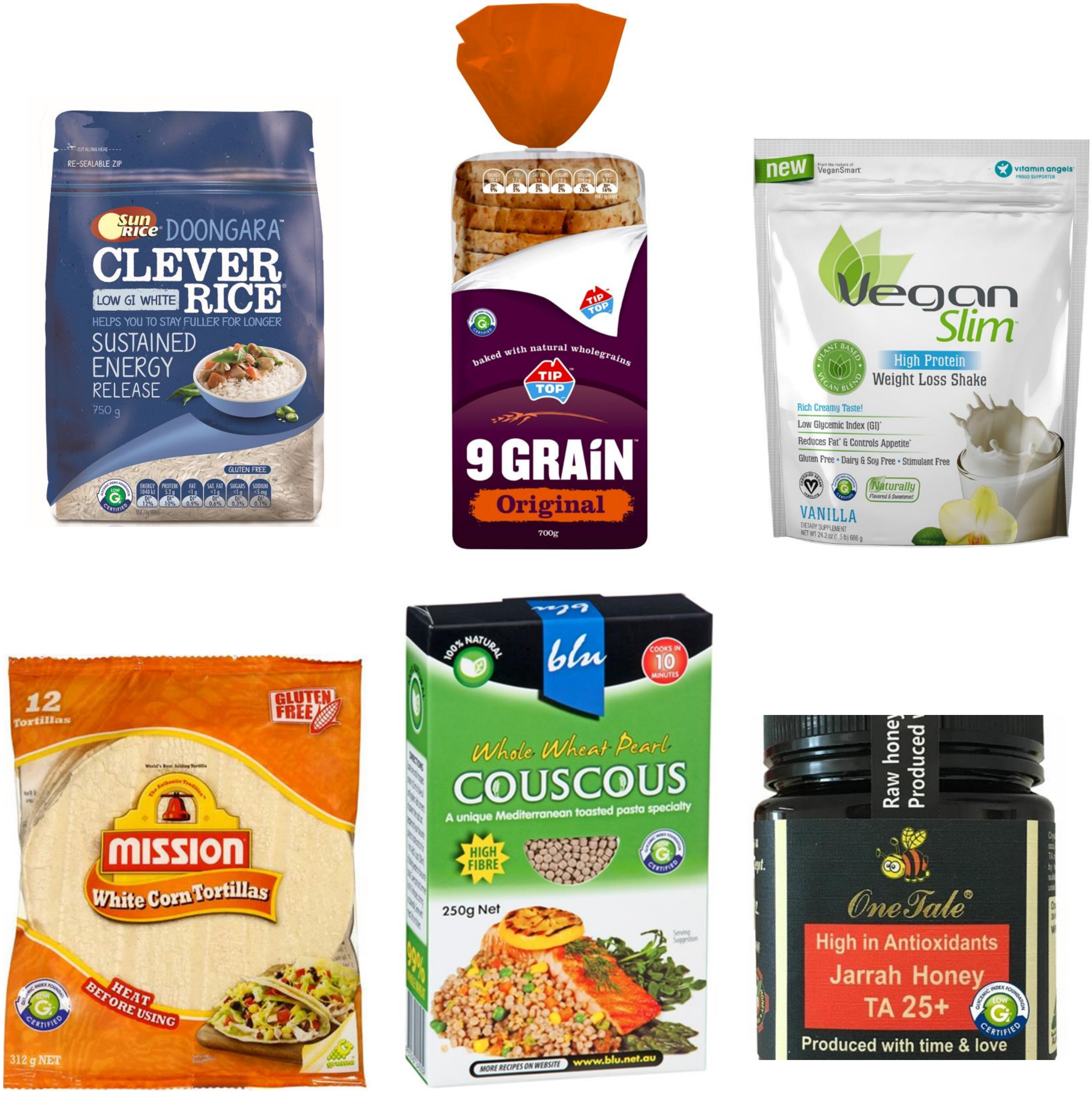

Supplementary Appendix 2C. Examples of foods with The Glycemic Index Foundation's Low GI Symbol shown to focus groups during Activity 3 [3].

References

1. The Glycemic Index Foundation. The GI Symbol Program. The Glycemic Index Foundation. https://www.gisymbol.com/ (october 4, 2018).

2. The Glycemic Index Foundation. Glycemic Index: The GI Symbol. The Glycemic Index Foundation. https://www.gisymbol.com/infographics/the-gi-symbol-infographic/ (October 10, 2018).

3. The Glycemic Index Foundation. Low GI Products. The Glycemic Index Foundation. https://www.gisymbol.com/products/ (October 10, 2018).
